# Slowness and Sparseness Have Diverging Effects on Complex Cell Learning

**DOI:** 10.1371/journal.pcbi.1003468

**Published:** 2014-03-06

**Authors:** Jörn-Philipp Lies, Ralf M. Häfner, Matthias Bethge

**Affiliations:** 1 Werner Reichardt Centre for Integrative Neuroscience, University of Tübingen, Tübingen, Germany; 2 Swartz Center for Theoretical Neurobiology, Brandeis University, Waltham, Massachusetts, United States of America; 3 Bernstein Center for Computational Neuroscience, Tübingen, Germany; 4 Max Planck Institute for Biological Cybernetics, Tübingen, Germany; Technische Universität Berlin, Germany

## Abstract

Following earlier studies which showed that a sparse coding principle may explain the receptive field properties of complex cells in primary visual cortex, it has been concluded that the same properties may be equally derived from a slowness principle. In contrast to this claim, we here show that slowness and sparsity drive the representations towards substantially different receptive field properties. To do so, we present complete sets of basis functions learned with *slow subspace analysis* (SSA) in case of natural movies as well as translations, rotations, and scalings of natural images. SSA directly parallels independent subspace analysis (ISA) with the only difference that SSA maximizes slowness instead of sparsity. We find a large discrepancy between the filter shapes learned with SSA and ISA. We argue that SSA can be understood as a generalization of the Fourier transform where the power spectrum corresponds to the maximally slow subspace energies in SSA. Finally, we investigate the trade-off between slowness and sparseness when combined in one objective function.

## Author Summary

A key question in visual neuroscience is how neural representations achieve invariance against appearance changes of objects. In particular, the invariance of complex cell responses in primary visual cortex against small translations is commonly interpreted as a signature of an invariant coding strategy possibly originating from an unsupervised learning principle. Various models have been proposed to explain the response properties of complex cells using a sparsity or a slowness criterion and it has been concluded that physiologically plausible receptive field properties can be derived from either criterion. Here, we show that the effect of the two objectives on the resulting receptive field properties is in fact very different. We conclude that slowness alone cannot explain the filter shapes of complex cells and discuss what kind of experimental measurements could help us to better asses the role of slowness and sparsity for complex cell representations.

## Introduction

The appearance of objects in an image can change dramatically depending on their pose, distance, and illumination. Learning representations that are invariant against such appearance changes can be viewed as an important preprocessing step which removes distracting variance from a data set in order to improve performance of downstream classifiers or regression estimators [1]. Clearly, it is an inherent part of training a classifier to make its response invariant against all within-class variations. Rather than learning these invariances for each object class individually, however, we observe that many transformations such as translation, rotation and scaling apply to any object independent of its specific shape. This suggests that signatures of such transformations exist in the spatio-temporal statistics of natural images which allow one to learn invariant representations in an unsupervised way.

Complex cells in primary visual cortex are commonly seen as building blocks for such invariant image representations (e.g. [2]). While complex cells, like simple cells, respond to edges of particular orientation they are less sensitive to the precise location of the edge [3]. A variety of neural algorithms have been proposed that aim at explaining the response properties of complex cells as components of an invariant representation that is optimized for the spatio-temporal statistics of the visual input [4]–[12].

The two main objectives used for the optimization of models of neural representations are *sparseness* and *slowness*. While in the context of unsupervised representation learning the two objectives have been proposed to similarly explain the receptive field properties of complex cells, there are important differences between them that may help to identify the algorithms used in biological vision. Intuitively, the slowness objective can be seen as a measure of approximate invariance or “tolerance”, whereas sparseness is better interpreted as a measure of selectivity. Tolerance and selectivity—or slowness and sparseness, respectively—can be understood as complementary goals which both play an important role for solving the task of object recognition [13]. A prominent view that goes back to Fukushima's proposal of the necognitron (1980) is that these goals are pursued in an alternating fashion by alternating layers of S and C cells where the S cells are optimized for selectivity and the C cells are optimized for tolerance. This idea has been inspired by the finding of simple and complex cells in primary visual cortex which also motivated the terminology of S and C cells.

Thus, based on the strong association between complex cells and invariance, one would expect that slowness rather than sparseness should play a critical role for complex cell representations. In this study, we investigate the differences between slowness and sparseness for shaping the receptive field properties of complex cells.

While for natural signals it may be impossible to find perfectly invariant representations, slowness seeks to find features that at least change as slowly as possible under the appearance transformations exhibited in the data [16], [9]–[12], [14]–[27]. In contrast to sparse representation learning which is tightly linked to generative modeling, many slow feature learning algorithms follow a discriminative or coarse-graining approach: they do not aim at modeling all variations in the sensory data but rather classify parts of it as noise (or some dimensions as being dominated by noise) and then discard this information. This is most obvious in the case of slow feature analysis (SFA) [21]. SFA can be seen as a special case of oriented principal component analysis which seeks to determine the most informative subspace under the assumption that fast changes are noise [28]. While it is very likely that some information is discarded along the visual pathway, throwing away information in modeling studies requires great caution. For example, if one discards all high spatial frequency information in natural images one would easily obtain a representation which changes more slowly in time. Yet, this improvement in slowness is not productive as high spatial frequency information in natural images cannot be equated with noise but often carries critical information. We therefore compare *complete* sets of filters learned with *slow subspace analysis* (SSA) [9] and *independent subspace analysis* (ISA) [4], respectively. The two algorithms are perfectly identical with the only difference that SSA maximizes slowness while ISA maximizes sparsity.

For sparseness it is common to show complete sets of filters, but this is not so in case of slowness. Based on the analysis of a small subset of filters, it has been argued that SSA may generally yield similar results to ISA [9]. In contrast, we here arrive at quite the opposite conclusion: by looking at the complete representation we find a large discrepancy between the filter shapes derived with SSA and those derived with ISA. Most notably, we find that SSA does not lead to localized receptive fields as has been claimed ([9], [29] —but see [28], [30]).

*Complete* representations optimizing slowness have previously been studied only for mixed objective functions that combined slowness with sparseness [8], [31]–[33] but never when optimizing exclusively for slowness alone. Here we systematically investigate how a complete set of filters changes when varying the objective function from a pure slowness objective to a pure sparsity objective by using a weighted mixture of the two and gradually increasing the ratio of their respective weights. From this analysis we will conclude that the receptive field shapes shown in [8], [31]–[33] are mostly determined by the sparsity objective rather than the slowness objective. That is the receptive fields would change relatively little if the slowness objective was dropped but it would change drastically if the sparsity objective was removed. These findings change our view of the effect of slowness and raise new questions that can guide us to a more profound understanding of unsupervised complex cell learning.

## Results

The central result of this paper is the observation that the effect of the slowness objective on complex cell learning is substantially different from that of sparseness. Most likely this has gone unnoticed to date because previous work either did not derive complete representations from slowness or combined the slowness objective with a sparsity constraint which masked the genuine effect of slowness. Therefore, we here put a large effort into characterizing the effect of slow subspace learning on the complete set of filter shapes under various conditions. We first study a number of analytically defined transformations such as translations, rotations, and scalings before we turn to natural movies and the comparison between slowness and sparseness.

**Figure 1:**
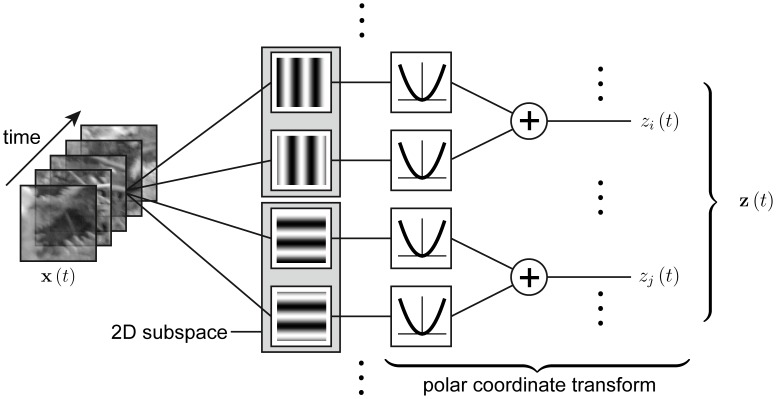
Model structure for both independent subspace analysis (ISA) and slow subspace analysis (SSA). The input signal, e.g. a movie sequence, is applied to several filters. Two filters form a subspace. The output of each filter is passed through a quadratic nonlinearity and summed within each subspace. The output corresponds to the radial component of the 2D subspace. The 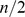 responses 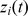 then form the multidimensional output signal 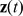. If the filters are the discrete Fourier transform basis where each subspace consists of the two filters which only differ in phase, then the output 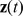 is the power spectrum of the input signal 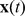.

The general design common to SSA and ISA is illustrated in Figure 1. We apply a set of filters to the input 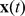 and square the filter responses. Two filters form a 2-dimensional subspace (gray box in Figure 1) and the sum of squared filter responses of these two filters yield the subspace energy response. This can be seen as the squared radial component of the projection of the signal into the 2D subspace formed by the two respective filters. For example, if the filters are taken from the Fourier basis and grouped such that the two filters within each subspace have the same spatial frequency and orientation and 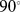 phase difference, the output 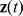 at a fixed time instant 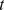 is the power spectrum of the image 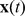. As input 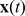 we used 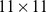 image patches sampled from the van Hateren image database [34] and from the video database [35], vectorized to 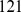-dimensions, and applied SSA to all remaining 120 AC components after projecting out the DC component.

**Figure 2:**
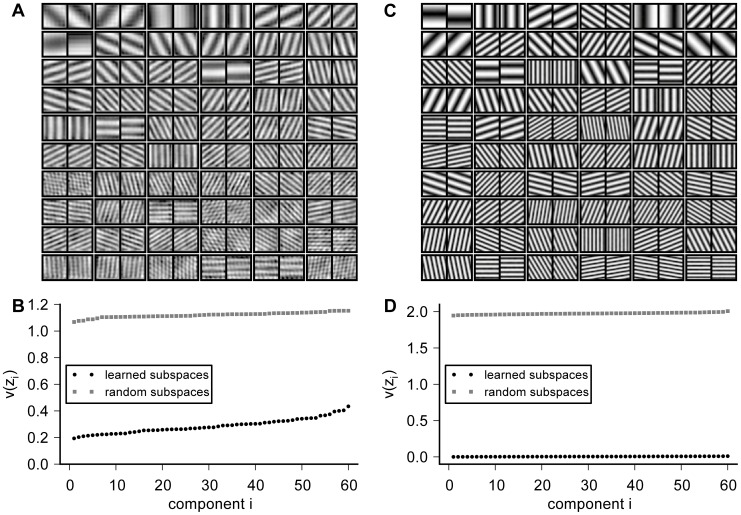
SSA on translations with open and cyclic boundary conditions. The complete set of filters learned from translated images with open and cyclic boundary conditions are shown in (A) and (C), respectively. Each row shows the filters of 6 subspaces with 2 dimensions. The subspaces are ordered according to their slowness, with the slowest filter in the upper left corner and decreasing slowness from left to right and top to bottom. The *inverse* slowness 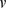 for the individual subspaces after learning (black dots) and for the initial random filters (gray squares) is shown in (B) and (D), respectively. For open boundary conditions (B), the inverse slowness does not converge to 0, hence perfect invariance is not achieved. For cyclic shifts, however, the inverse slowness approaches 0 with arbitrary precision (D), indicating convergence to perfect invariance.

In the first part of our study, the input sequence consisted of translations. As time-varying process for the translations, we implemented a two-dimensional random walk of an 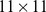 window over the full image. The shift amplitudes were drawn from a continuous uniform distribution between 0 and 2 pixels, allowing for subpixel shifts. The filters obtained from SSA are shown in Figure 2A. Each row contains the filter pairs of 6 subspaces, sorted by descending slowness from left to right and top to bottom. The filters clearly resemble global sine wave functions. The wave functions differ in spatial frequency and orientation between the different subspaces. Within each subspace, orientation and spatial frequency are almost identical, but phases differ significantly. In fact, the phase difference is close to 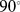 (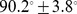), resembling quadrature pairs of sine and cosine functions as it is the case for the two-dimensional Fourier basis. Accordingly, the subspace energy output 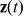 of the resulting SSA representation is very similar to the power spectrum of the image 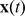.

In fact, one can think of SSA as learning a generalized power spectrum based on a slowness criterion. While the power spectrum is known to be invariant against translations with periodic boundary conditions, perfect invariance—or infinite slowness—is not achieved for the translations with open boundary conditions studied here (see Figure 2 B). The slowness criterion is best understood as a penalty of fast changes since it decomposes into an average over penalties of fast changes for each individual component (see methods). Therefore, we will always show the inverse slowness 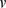 for each component such that the *smaller* the area under the curve the *better* the average slowness.

Compared to random subspaces, the decrease in 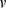, i.e. the increase in slowness, is substantial: the average inverse slowness 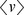 decreases approximately by a factor of three. The low frequency subspaces are clearly the slowest subspaces, and slowness decreases with increasing spatial frequency. At the same time, however, the inverse slowness of all learned subspaces is still larger than 0, i.e. even for the slowest components, perfect invariance is not achieved. This is not surprising, as perfect invariance is impossible whenever unpredictable variations exist as it is the case for open boundary conditions.

In Figure 2 C, we show that SSA can indeed find perfectly invariant filters starting from a random initial filter set if one imposes periodic boundary conditions. To this end, we created 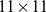 pink noise patches with circulant covariance structure, i.e. the pixels on the left border of the image are correlated with pixels on the right border as if they were direct neighbors. As time-varying process, we implemented a random walk with cyclic shifts where the patches were translated randomly with periodic boundary conditions. As in the previous study, the shift amplitudes were drawn from a continuous uniform distribution between 0 and 2 pixels. Since the Fourier basis is the eigenbasis of the cyclic shift operator it should yield infinite slowness for the cyclic boundary conditions. Indeed, the filters learned from these data recover the Fourier basis with arbitrary precision. Perfect invariance is equivalent with the objective function converging to 0. This means that the response of each subspace is identical for all shifts. Figure 2D shows the inverse slowness 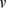 of the individual components. For all filters, 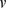 is very small (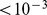), close to perfect invariance and infinite slowness.

**Figure 3:**
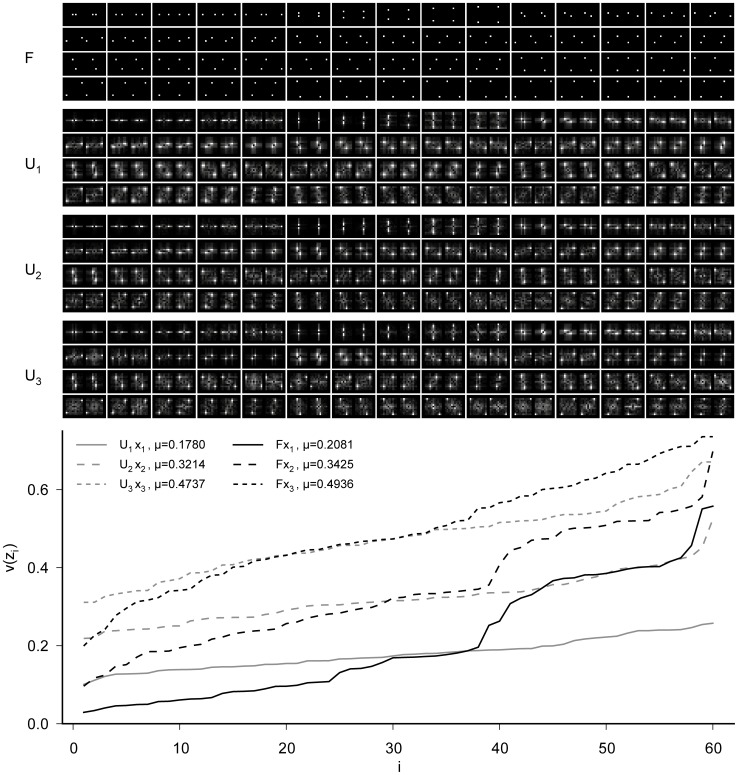
Deviations from the Fourier basis for translations with open boundary conditions. Here, we started the optimization with the Fourier basis (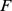) as initial condition. We used 3 different data sets sampled from the van Hateren image database using 2D translations with a shift amplitude of maximally 1, 2, or 3 pixels. The optimized filters 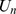, where 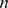 is the maximal shift amplitude, do not deviate dramatically from the initial condition. The amplitude spectra of all filters are shown in the *upper panel* with the DC component being at the center. The amplitude spectra of the optimized filters blur out towards the lower frequencies except for the lowest frequencies, which blur out towards the higher frequencies. Only the highest frequencies show additional sensitivity at the lowest spatial frequencies which cannot be explained by spatial localization. The slowness of the individual components is shown in the *lower panel*. The black lines indicate the performance of the Fourier basis applied to test data with shift amplitudes of up to 1 (solid), 2 (long dashes), or 3 (short dashes) pixels. The gray lines show the performance of the optimal filters. SSA sacrifices slowness on the slower filters to gain a comparatively larger amount of slowness on the faster filters. In this way, overall SSA achieves better slowness.

Given that the SSA representation learned for translations is very similar to the Fourier basis and since the Fourier basis achieves perfect invariance for cyclic shifts we proceeded to investigate whether the Fourier basis is optimal even for non-cyclic translations as well. We created three different data sets, with random translations as in the first study, but the maximal shift amplitude of the 2D random walk was 1, 2, and 3 pixels, respectively. As initial condition, we used the Fourier basis (Figure 3, ‘
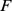
’) instead of a random matrix. The optimized bases are denoted as 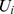 where 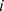 indicates the maximal shift amplitude. We show the 2D-Fourier amplitude spectrum of the filters rather than the filters in pixel space because it is easier to assess the differences between the different bases. The DC component is located at the center of the spectrum.

During optimization, the basis slightly departs from the initial condition but remains very localized in the Fourier domain (Figure 3, ‘
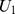
’). The low frequency filters become sensitive to higher frequencies while the high frequency filters become also sensitive to lower frequencies as the initial filters blur out towards the border or center, respectively. The objective function is improved for the optimized filters not only on the training but also on the test set (cf. Table 1). The slowness of the 60 individual components 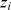 evaluated on identically created test sets (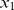, 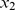, and 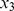, respectively) is shown in Figure 3. The Fourier filters are slower than the optimized filters for the first 20–30 components, then about equal for 10 components, and significantly faster for the remaining components. Apparently, the SSA objective sacrifices a little bit of the slowness of the low frequency components to get a comparatively larger gain in slowness from modifying the high frequency components. The optimization of average inverse slowness in contrast to searching for a single maximally slow component is a characteristic feature of SSA.

**Table 1:** Control for overfitting.

	Fourier basis		optimized basis	
	training	test	training	test
1 pixel shift	0.17838	0.17725	0.13801	0.15359
2 pixel shift	0.29469	0.29185	0.24680	0.27570
3 pixel shift	0.41521	0.41943	0.36569	0.40423

Objective on training and test set for optimized filters and Fourier basis.

**Figure 4:**
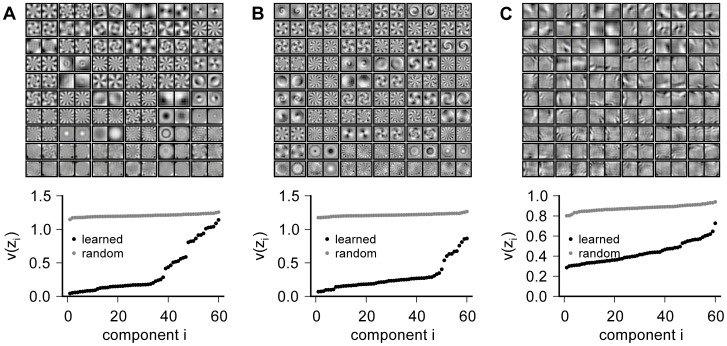
SSA filters for local rotation and scaling. Illustration of the filters obtained from patch-centered rotation sequences (A,B) and patch-centered scaling sequences (C) with the slowness of the individual filter subspaces before (*random*) and after the optimization (*learned*). The filters are ordered in ascending inverse slowness 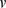 (row-wise) with the slowest feature in the upper left and the fastest feature in the lower right corner. The data in (A) and (C) consist of 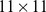 square patches from the van Hateren data set while the data for (B) consist of 121-dimensional round patches which are, for visualization, embedded in a 14×14 square patch. The rotation filters match those found in steerable filter theory [28]. The filters of the patch-centered anisotropic scaling exhibit localized edge filters centered towards the patch boundaries.

Even though we expect changes in natural movies to be dominated by local translations, it is instructive to study other global affine transforms as well. Therefore, we applied SSA to 3 additional data sets: The first data set contains 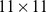 patches from the van Hateren image set which were rotated around the center pixel. The second data set consists of 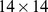 patches from the van Hateren image set which were also rotated around the center pixel but where we kept only the pixels within a predefined circle. Specifically, we reduced the number of dimensions again to 121 pixels by cutting out the corners which left an 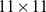 circular image patch. The patches in the third data set were sampled with sizes ranging from 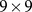 to 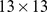 pixels and then rescaled to 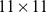 pixels, in order to obtain a patch-centered anisotropic scaling transformation. The preprocessing was identical to the previous studies and the initial filter matrix was a random orthonormal matrix. The filters and the objective of the individual subspaces of the 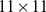 rotation data are shown in Figure 4A. The filters resemble the rotation filters found with steerable filter theory [28]. The slowness of all components is significantly larger than for random filters, but with clearly decreasing slowness for the last subspaces. Notably, the last subspaces have no systematic structure. This can be explained by the fact that when rotating a square patch, the pixels in the 4 corners are not predictable unless for multiples of 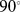 rotations. Therefore the algorithm cannot find meaningful subspaces that would preserve the energy for the pixels in the corners. The filters in Figure 4B from the disc shaped patches do not show these artifacts. Here, all filters nicely resemble angular wave functions as expected from steerable filter theory and also exhibit better slowness. Finally, the scaling filters are shown in Figure 4C. All filters resemble windowed wave functions that are localized towards the boundaries of the patch. This indicates that a scaling can be seen as a combination of local translations which go inward for downscaling and outward for upscaling. All subspaces defined by the learned filters are significantly slower than the random subspaces.

**Figure 5:**
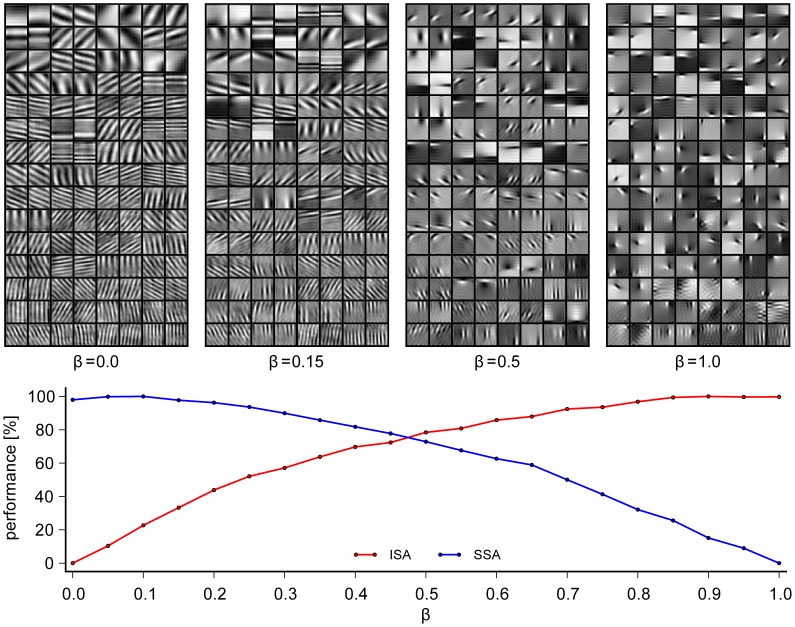
Filters of slowness, independence and mixture objective learned on movies. The lower panel shows the performance with respect to both the slowness objective 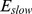 (blue) and the sparsity objective 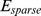 (red) and the upper panel displays four sets of filters as obtained for different values for the trade-off parameter 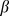: The leftmost case (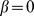) is equivalent to SSA and the rightmost case (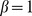) is equivalent to ISA. There is a large difference between the two that can easily be grasped by eye. The example for 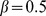 reflects the crossing point in performance (see lower panel) meaning that the representation performs slightly better than 80% of its maximal performance with respect to both objectives simultaneously. The case 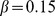 was hand-picked to represent the point where the filters perceptually look similarly close to ISA and SSA.

After characterizing the result of slow subspace learning for analytically defined transformations we now turn to natural movies and the comparison between slowness and sparseness. Specifically, we compare slow subspace analysis (SSA) to independent subspace analysis (ISA) in order to show how the slowness and the sparsity objective have different effects on the receptive field shapes learned. To this end, we combine the two objectives to obtain a weighted mixture of them for which we can gradually tune the trade-off between the slowness and the sparseness objective. In this way, we obtain a 1-parametric family of objective functions
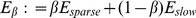
(1)for which the parameter 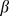 determines the trade-off between slowness and sparseness. Specifically, we obtain SSA in case of 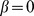 and ISA for 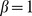. As one can see in Figures 5 the filters learned with SSA (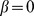) look very different from those learned with ISA (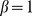). This finding contradicts earlier claims that the filters learned with SSA are comparable to those learned with ISA. The most obvious difference is that the slowness objective works against the localization of filters that is brought forward by the sparsity objective.

For 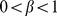 we will refer to the resulting algorithm as *independent slow subspace analysis* (ISSA). If a representation is optimized for 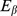 its performance with respect to the slowness objective 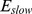 decreases monotonically with 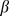. At the same time, its performance with respect to 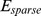 increases with 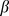. The percentages shown indicate the increase in slowness and sparseness relative to the maximal gain that can be achieved if one optimizes solely for one of the two objectives. Note that the shapes of these curves depend on the objective functions used and are not invariant under pointwise nonlinear transformations. The values shown here are determined directly by the objective functions without any additional transformation (see Eqs. 3,11). Remarkably, it is possible to derive a representation which performs reasonably well with respect to both sparseness and slowness simultaneously. At an intermediate point where both objectives, 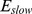 and 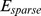, are reduced by the same factor in our units, the performance is still larger than 80% for each. Interestingly, for this trade-off the receptive fields look quite similar to those obtained with ISA. This may explain why previous work on unsupervised learning with combinations of sparseness and slowness did not reveal that the two objectives drive the receptive fields towards very different shapes.

**Figure 6:**
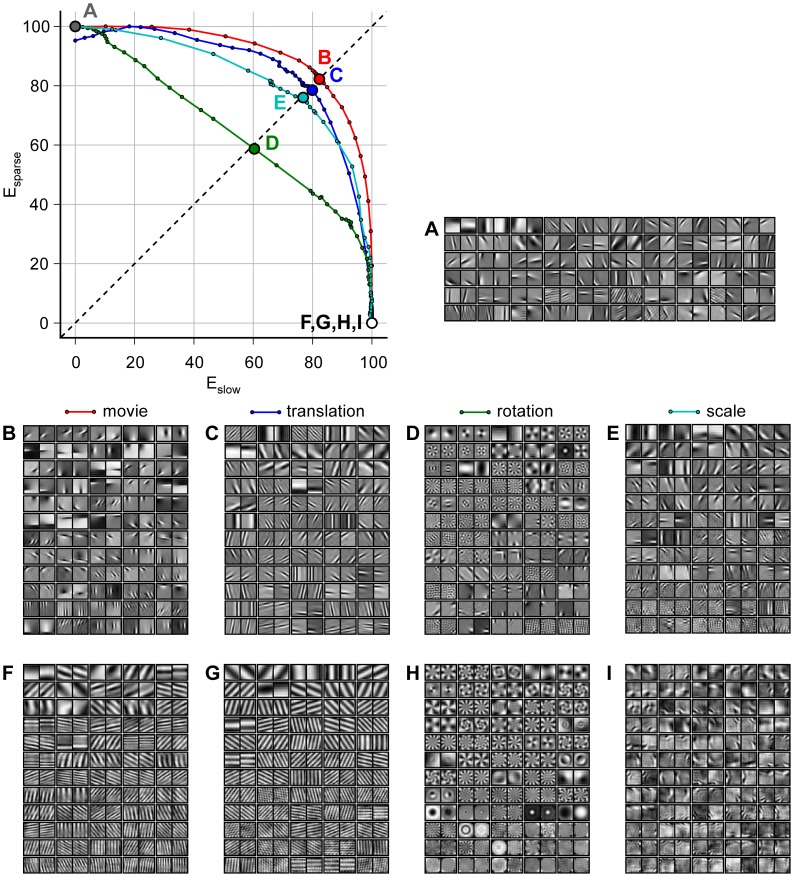
Trade-off in the performance with respect to slowness and sparsity. When optimizing the filter set for a weighted superposition of the slowness and sparsity objectives the performance with respect to 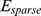 decreases monotonically with 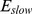 (*upper left*). The steepness of decay indicates the impact of the trade-off. The different colors correspond to different datasets (see legend). While the performance with respect to 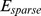 for the rotation data falls off quickly (green), the differences between scaling, translation and movie data (cyan, blue, red) are not significant. The concave shapes of the curves indicate a rather gentle trade-off. The dashed diagonal line indicates an intermediate point for this trade-off. We chose it such that both objectives are reduced by the same factor relative to their optimal performance in the units used here. The corresponding filters are shown in the adjacent panels: The ISA filters are shown in (A) which are independent of the temporal statistics. The ISSA filters at the break even point are shown in (B) for movies, in (C) for translations, in (D) for rotations, and in (E) for scalings. The last row shows the SSA filters in the same order: (F) for movies, in (G) for translations, in (H) for rotations, and in (I) for scalings.

The trade-off in performance with respect to slowness and sparsity for natural movies, translation, rotation, and scaling is summarized in Figure 6. It shows the ISA filters (A), the ISSA filters at the intermediate point of slowness and sparsity for natural movies (B), translation (C), rotation (D), and scaling (E) and in the same order the SSA filters in (F,G,H,I). The concave shape of the curves (upper left) indicates that the trade-off between the two objectives is rather graceful such that it is possible to achieve a reasonably good performance for both objectives at the same time.

## Discussion

Unsupervised learning algorithms are a widespread approach to study candidate computational principles that may underly the formation of neural representations in sensory systems. Slowness and sparsity both have been suggested as objectives driving the formation of complex cell representations. More specifically, it has been claimed that the filter properties obtained from slow subspace analysis would resemble those obtained with independent subspace analysis [9] and that the optimal stimulus for SFA is localized [29]. Here, we showed that there is a striking difference between the sets of SSA and ISA filters: While the sparsity objective of ISA facilitates localized filter shapes, maximal slowness can be achieved only with global receptive fields as found by SSA.

The different implications of slowness and sparseness are most notable in filters containing high spatial frequencies. For low spatial frequency filters the number of cycles is small simply because it is constrained to be smaller than the product of spatial frequency and simulation window size. Since previous studies have inspected only low spatial frequency filters the different effect of sparseness and slowness has gone unnoticed or at least not been sufficiently appreciated [6], [9], [29]. A signature of the drive towards global filters generated by slowness can be found in the bandwidth statistics presented in [6]. Global filter shapes correspond to small bandwidth. While the authors mention that the fraction of small bandwidth filters exceeds that found for physiological receptive fields they rather suggested that this may be an artifact of their preprocessing, specifically referring to dimensionality reduction based on principal component analysis. However, the opposite is the case: the preprocessing rather leads to an *underestimation* of the fraction of small bandwidth filters. Principal component analysis will always select for low spatial frequency components and thus reduce the fraction of small bandwidth filters because it is the high spatial frequency components which have the smallest bandwidth.

While it is difficult to make rigorous statements that are model-independent, there are general arguments why the lack of localization is likely a generic consequence of slowness rather than a spurious property that was specific to SSA only: By definition a neuron cannot be driven by stimuli outside of its receptive field (RF). Therefore, whenever a stimulus is presented that drives the neuron inside its RF, the neuron must stop firing when the stimulus is shifted outside the RF. This suggests very generally, that in the presence of motion the objective of slowness or invariance necessarily requires large RFs. Sparsity, in contrast, encourages neurons to respond as selectively as possible. One obvious way to achieve this is to become selective for location which directly translates into small RF sizes.

In addition, analytical considerations suggest that slowness is likely to generate global filters with small bandwidth. For small image patches it is reasonable to assume that the spatio-temporal statistics are dominated by translational motion. Thus, it is not surprising that the filter properties of SSA found for natural movies resemble those for translations. In computer vision, there is a large number of studies which derive features that are invariant under specific types of transformations such as translations, scalings and rotations. An analytical approach to invariance is provided by steerable filter theory [36], [37] which allows one to design perfectly invariant filters for any compact Lie group transformation [38]. The best known example is the power spectrum which is perfectly invariant under translations with periodic boundary conditions [28]. For the other Lie group transformations studied in this paper, the symmetry was broken due to discretization and boundary effects. In these cases the representations found with SSA can be seen as a generalization of the Fourier transform whose subspace energies are not perfectly invariant anymore but at least maximally stable under the given spatio-temporal statistics. A very similar argument has also been made for SFA [30].

The receptive fields of complex cells determined from physiological experiments rarely exhibit multiple cycles as predicted by SSA. This indicates that complex cells in the brain are not fully optimized for slowness. It may still be possible though that slowness plays some role in the formation of complex cells. The trade-off analysis with the mixed objective has shown that giving up some sparsity allows one to achieve both relatively large sparsity and slowness at the same time with localized receptive fields.

Having established how exactly sparseness and slowness differ in their implied receptive fields also helps to address the roles of sparseness and slowness experimentally. Li & DiCarlo [39], [40] found neural correlates of the learning of invariances by manipulating the statistics of the presented stimuli. Since their recordings were from area IT where receptive fields are known to be very large, it would be very interesting to see the effect of similar experiments, made during the critical period, on complex cells in primary visual cortex. To distinguish between slowness and sparseness it might also be instructive to vary the temporal continuity of the training stimuli, e.g. by comparing the effect of smooth translations with discrete jumps on the learnt receptive fields. Another, possibly more direct approach to distinguish between sparseness and slowness might be to compute the respective objective functions directly on the sensory responses over development. While such an experiment has already been done for sparseness by [8] who interestingly found that sparseness *decreases* throughout development, we are not aware of the equivalent evaluation of any change in neuronal slowness.

Independent of what happens during development, the comparison of slowness and sparseness raises questions about how we should view the role of complex cells with respect to the tolerance-selectivity trade-off. Given that large receptive fields are advantageous for invariance or slowness, the small receptive field size of complex cells suggests that complex cells do not aim at achieving maximal tolerance but rather lean towards preserving a high degree of selectivity. For both ISA and SSA some degree of invariance is already built into the architecture which resembles the energy model of complex cells and will always find two-dimensional invariant subspaces. Instead of prescribing the invariant subspace dimensionality we wanted to know what happens if the subspace dimensionality is learned as well. This can be done by learning complex cells with SFA on the full quadratic feature space and then investigating the spectrum of the resulting quadratic forms. Comparing the number of subspaces employed by SFA to maximize slowness to empirical measurements in V1 [41], [42] it turns out that the number of subspaces employed by real neurons, and therefore the degree of invariance is smaller than predicted by slowness (see Figure S1).

The deeper principle underlying both sparsity and slowness is the idea of generative modeling [25]. From a generative modeling perspective, one is most concerned about modeling the precise shape of all variations in the data rather than just optimizing some fixed architecture or feature space to be as invariant or sparse as possible. More specifically, in a generative modeling framework all ingredients of the model are formalized by a density model and thus the likelihood becomes the natural objective function. This holds also true for the studies which combined the slowness objective with a sparsity objective in the past [8], [31]–[33]. The generative power of these models, however, still needs to be significantly improved in order to be able to explain object recognition performance of humans and animals. A better understanding of the partially opposing demands of slowness and sparseness on the response properties of visual neurons will help us understand the computational strategy employed by the visual system in reaching that performance.

## Methods

### Slow Subspace Analysis

The algorithm of slow subspace analysis (SSA) has previously been described by Kayser et al [9]. Just like in independent subspace analysis [4] also in SSA the 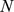-dimensional input space is separated into 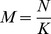 independent subspaces of dimensionality 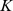 and the (squared) norm of each subspace should vary as slowly as possible. The output function of the 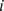-th subspace is then defined as
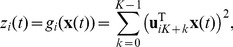
(2)where K is the dimensionality of the subspace, 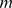 the number of the subspace, and 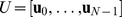 is the orthonormal filter matrix. It is important to notice that, for an input signal 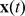 with zero mean and unit variance, 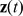 has mean 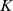. For 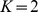, the set of squared subspace norms corresponds to the power spectrum of the Fourier transform if the set of filters are the discrete Fourier transform.

The objective function of SSA has been called “temporal smoothness” objective by Kayser *et al.* [9] and is given by
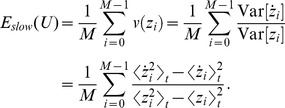
(3)Note, however, that 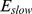 increases with the amount of rapid changes and is minimized subject to 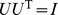. To find the optimal set of filters 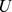 under the given constraints we use a variant of the gradient projection method of Rosen [43] which was successfully used for simple cell learning before [22].

In order to compute the gradient of the objective function we have to compute the temporal derivative of the output signal 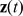 first, using the difference quotient as approximation:
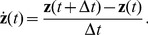
(4)As we use discrete time steps, we can set 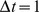 which leads to 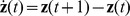. This simplifies the objective function (3) as the temporal difference mean 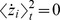. The objective function can be further simplified by using the fact that 
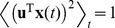
 for 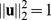 and 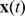 having zero mean and unit variance, which leads to 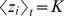. The complete objective function is then
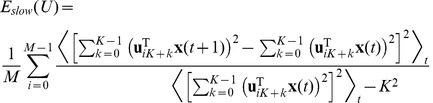
(5)For every iteration, the gradient of the objective function is computed, scaled by the step length 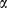, and subtracted from the current filter set
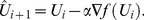
(6)The partial gradient with respect to 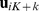 is
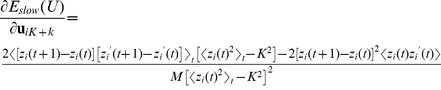
(7)with
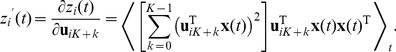
(8)The matrix containing the resulting filter set is then projected onto the orthogonal group using symmetric orthogonalization [44]

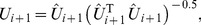
(9)yielding the closest orthonormal matrix with respect to the Frobenius norm [45]. Along this gradient a line search is performed where the initial step length 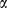 is reduced until the objective function on 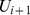 is smaller than 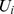 before the iteration proceeds.

The optimization is initialized with a random orthonormal matrix 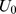. As stopping criterion the optimization terminates when the change in the objective function is smaller than the threshold 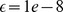. In all our simulations we used a subspace dimension of 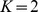. A python implementation of the algorithm can be found as part of the natter toolbox http://bethgelab.org/software/natter/.

### Independent Subspace Analysis

Independent subspace analysis (ISA) has originally been proposed by Hyvärinen and Hoyer [4]. The only difference between SSA and ISA is the objective function. Generally speaking, ISA is characterized by a density model for which the density factorizes over a decomposition of linear subspaces. In most cases the subspaces all have the same dimension, and in case of natural images the marginal distributions over the individual subspaces are modeled as sparse spherically symmetric distributions. Like Hyvärinen and Hoyer [4] we chose the spherical exponential distribution
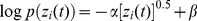
(10)where 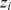 is the subspace response as defined in Equation 2, 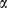 is a scaling constant and 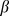 the normalization constant. Correspondingly, the objective function reads
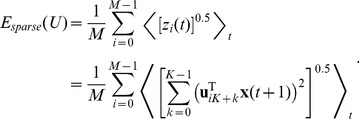
(11)The scaling and normalization constants 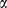 and 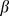 can be omitted. This leads to the gradient
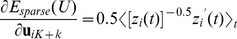
(12)with 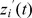 as defined in Equation 8. The optimization is identical to SSA where only objective and gradient are replaced. For the numerical implementation of ISA we used a python translation of the code provided by the original authors at http://research.ics.aalto.fi/ica/imageica/.

### Data Collection

The time-varying input signal 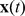 was derived from the van Hateren image database [34] for translations, rotations and scalings and the van Hateren movie database [35] for movie sequences. The image database contains over 4000 calibrated monochrome images of 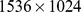 pixels, where each pixel corresponds to 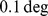 of visual angle. We created a temporal sequence by sliding a 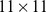 window over the image. Step length and direction for translation, angle for rotation and anisotropic scaling factors were sampled from a uniform random process. If not stated otherwise, the translation was sampled independently for x- and y direction from a uniform distribution on 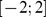, the rotation angle from a uniform distribution on 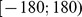 and the scaling factors independently for x- and y-direction from a uniform distribution on 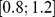. The movie database consists of 216 movies of 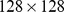 pixels with a duration of 192 s and 25 frames per second. The images were taken in Holland and show the landscape consisting mostly of bushes, trees and lakes with the occasional streets and houses. The video clips were recorded from Dutch, German and British television with mostly wildlife scenes but also sports and movies. For each stimulus set we sampled 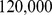 patches.

### Preprocessing

The extracted 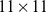 image patches were treated as vectors by stacking up the columns of the image patches, resulting in a 121-dimensional input vector 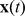. We projected out the DC component, i.e. removed the mean from the patches, and applied symmetric whitening to the remaining 120 AC components. No low pass filtering or further dimensionality reduction was applied. All computations were done in the 120-dimensional whitened space and the optimized filters then projected back into the original pixel space.

## Supporting Information

Figure S1
**Model complex cells derived with SFA fail to reproduce the small numbers of significant eigenvalues found empirically with STC analysis.** We computed SFA filters on the quadratic feature space of the 100 lowest Fourier components of 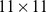 image patches sampled from the van Hateren image database [34]. As temporal transformation we applied a 2D translation with shift amplitudes drawn from a 2D uniform continuous distribution on 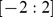 to the data. We then applied the same analysis to the SFA filters as used in [42], [41]. We applied a sequence of 50000 Gaussian white noise pattern to the SFA filter. The filter responses were centered at their respective median and split in two firing rate sets, the excitatory from all positive responses (i.e. larger than median) and the inhibitory from the absolute value of all negative responses (i.e. smaller than median). The firing rates were then used to generate Poisson spike counts. Given spike counts and stimuli, we computed the spike triggered covariance (STC) for 100 different noise stimulus sets per SFA filter. The spectrum of eigenvalues (eigenspectrum) of the STC matrix of one cell recorded from V1 in an awake monkey [41] is shown in (A), the eigenspectrum of the STC of one SFA filter is shown in (B). To determine which eigenvectors are significant, we computed the STC with shuffled spike counts as control. The dashed lines correspond to mean 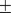 4.4 SD, which corresponds to a confidence interval of 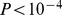 for Gaussian distributed eigenvalues. One clear difference is the number of significant eigenvectors. While for the V1 cell, only a few eigenvectors are significant, for the SFA model almost all eigenvectors are significant. The histogram of the number of significant excitatory and inhibitory eigenvectors is shown in (C) for the physiological data and in (D) for the SFA model. While the V1 cells have only few significant eigenvectors for all 130 recorded cells, the 980000 cells of the SFA model have on average 80 significant excitatory and inhibitory eigenvectors out of the 100 dimensions. The histogram bins with 0 entries were not plotted for clarity of the figure.(PDF)

## References

[1] Burges CJC (2005) Geometric Methods for Feature Extraction and Dimensional Reduction. In: Maimon O, Rokach L, editors, Data Mining and Knowledge Discovery Handbook: A Complete Guide for Practitioners and Researchers, Kluwer Academic Publishers. pp. 59–92.

[2] Riesenhuber M, Poggio T (1999) Hierarchical models of object recognition in cortex. Nature Neuroscience 2: 1019–25. 10526343 10.1038/14819

[3] Hubel DH, Wiesel TN (1962) Receptive fields, binocular interaction and functional architecture in the cat's visual cortex. The Journal of Physiology 160: 106–154. 14449617 10.1113/jphysiol.1962.sp006837 PMC1359523

[4] Hyvärinen A, Hoyer P (2000) Emergence of phase-and shift-invariant features by decomposition of natural images into independent feature subspaces. Neural Computation 12: 1705–1720. 10935923 10.1162/089976600300015312

[5] Hyvärinen A, Karhunen J, Oja E (2001) Independent Component Analysis. New York, NY, USA: John Wiley & Sons, Inc., 481 pp.

[6] Berkes P, Wiskott L (2005) Slow feature analysis yields a rich repertoire of complex cell properties. Journal of Vision 5: 579–602. 16097870 10.1167/5.6.9

[7] Karklin Y, Lewicki MS (2009) Emergence of complex cell properties by learning to generalize in natural scenes. Nature 457: 83–86. 19020501 10.1038/nature07481

[8] Berkes P, Turner RE, Sahani M (2009) A Structured Model of Video Reproduces Primary Visual Cortical Organisation. PLoS Computational Biology 5: 16. 10.1371/journal.pcbi.1000495 PMC2726939 19730679

[9] Kayser C, Einhäuser W, Dümmer O, König P, Körding KP (2001) Extracting Slow Subspaces from Natural Videos Leads to Complex Cells. In: Artificial Neural Networks - ICANN 2001. Austrian Res Inst Artifical Intelligence, volume 2130, pp. 1075–1080. doi:10.1007/3-540-44668-0149.

[10] Einhäuser W, Kayser C, König P, Körding KP (2002) Learning the invariance properties of complex cells from their responses to natural stimuli. European Journal of Neuroscience 15: 475–486. 11876775 10.1046/j.0953-816x.2001.01885.x

[11] Kayser C, Körding KP, König P (2003) Learning the nonlinearity of neurons from natural visual stimuli. Neural Computation 15: 1751–9. 14511511 10.1162/08997660360675026

[12] Körding KP, Kayser C, Einhäuser W, König P (2004) How are complex cell properties adapted to the statistics of natural stimuli? Journal of Neurophysiology 91: 206–212. 12904330 10.1152/jn.00149.2003

[13] DiCarlo JJ, Zoccolan D, Rust NC (2012) How does the brain solve visual object recognition? Neuron 73: 415–34. 22325196 10.1016/j.neuron.2012.01.010 PMC3306444

[14] Sutton RS, Barto AG (1981) An adaptive network that constructs and uses an internal model of its world. Cognition and Brain Theory 4: 217–246.

[15] Klopf AH (1982) The Hedonistic Neuron: A Theory of Memory, Learning, and Intelligence. Washington DC: Hemisphere Publishing Corporation, 140 pp.

[16] Földiák P (1991) Learning Invariance from Transformation Sequences. Neural Computation 3: 194–200. 10.1162/neco.1991.3.2.194 31167302

[17] Mitchison G (1991) Removing Time Variation with the Anti-Hebbian Differential Synapse. Neural Computation 3: 312–320. 10.1162/neco.1991.3.3.312 31167313

[18] Stone JV, Bray A (1995) A learning rule for extracting spatio-temporal invariances. Network Computation in Neural Systems 6: 429–436.

[19] Stone JV (1996) Learning Perceptually Salient Visual Parameters Using Spatiotemporal Smoothness Constraints. Neural Computation 8: 1463–1492. 8823943 10.1162/neco.1996.8.7.1463

[20] Wallis G, Rolls ET (1997) A model of invariant object recognition in the visual system. Progress in Neurobiology 51: 167–194. 9247963 10.1016/s0301-0082(96)00054-8

[21] Wiskott L, Sejnowski TJ (2002) Slow feature analysis: Unsupervised learning of invariances. Neural computation 14: 715–770. 11936959 10.1162/089976602317318938

[22] Hurri J, Hyvärinen A (2003) Simple-cell-like receptive fields maximize temporal coherence in natural video. Neural Computation 15: 663–91. 12620162 10.1162/089976603321192121

[23] Spratling MW (2005) Learning viewpoint invariant perceptual representations from cluttered images. Pattern Analysis and Machine Intelligence, IEEE Transactions on 27: 753–61. 10.1109/TPAMI.2005.105 15875796

[24] Maurer A (2006) Unsupervised slow subspace-learning from stationary processes. In: Proceedings of the 17th international conference on Algorithmic Learning Theory. Berlin, Heidelberg: SpringerVerlag, volume 4264 of *Lecture Notes in Computer Science*, pp. 363–377.

[25] Turner R, Sahani M (2007) A Maximum-Likelihood Interpretation for Slow Feature Analysis. Neural Computation 19: 1022–38. 17348772 10.1162/neco.2007.19.4.1022

[26] Masquelier T, Serre T, Poggio T (2007) Learning complex cell invariance from natural videos: A plausibility proof. Technical report, Massachusetts Institute of Technology Computer Science and Artifificial Intelligence Laboratory.

[27] Maurer A (2008) Unsupervised slow subspace-learning from stationary processes. Theoretical Computer Science 405: 237–255.

[28] Bethge M, Gerwinn S, Macke JH (2007) Unsupervised learning of a steerable basis for invariant image representations. In: Proceedings of SPIE Human Vision and Electronic Imaging XII (EI105). volume 6492, p. 12.

[29] Wiskott L, Berkes P, Franzius M, Sprekeler H, Wilbert N (2011) Slow feature analysis. Scholarpedia 6: 5282, revision #136882.

[30] Sprekeler H, Wiskott L (2011) A theory of slow feature analysis for transformation-based input signals with an application to complex cells. Neural Computation 23: 303–335. 21105830 10.1162/NECO_a_00072

[31] Hyvärinen A, Hurri J, Väyrynen J (2003) Bubbles: a unifying framework for low-level statistical properties of natural image sequences. Journal of the Optical Society of America A 20: 1237–1252. 10.1364/josaa.20.001237 12868630

[32] Cadieu C, Olshausen BA (2009) Learning transformational invariants from natural movies. Advances in Neural Information Processing Systems 21: 209–216.

[33] Cadieu C, Olshausen BA (2012) Learning intermediate-level representations of form and motion from natural movies. Neural Computation 24: 827–66. 22168556 10.1162/NECO_a_00247

[34] van Hateren JH, van der Schaaf A (1998) Independent component filters of natural images compared with simple cells in primary visual cortex. Proceedings of the Royal Society B: Biological Sciences 265: 359–366. 9523437 10.1098/rspb.1998.0303 PMC1688904

[35] van Hateren JH, Ruderman DL (1998) Independent component analysis of natural image sequences yields spatio-temporal filters similar to simple cells in primary visual cortex. Proceedings of the Royal Society B: Biological Sciences 265: 2315–20. 9881476 10.1098/rspb.1998.0577 PMC1689525

[36] Knutsson H, Granlund GH (1983) Texture Analysis Using Two-Dimensional Quadrature Filters. In: IEEE Computer Society Workshop on Computer Architecture for Pattern Analysis and Image Database Management. pp. 206–213.

[37] Freeman WT, Adelson EH (1991) The design and use of steerable filters. IEEE Transactions on Pattern analysis and machine intelligence 13: 891–906.

[38] Hel-Or Y, Teo PC (1998) Canonical decomposition of steerable functions. Journal of Mathematical Imaging and Vision 9: 83–95.

[39] Li N, DiCarlo JJ (2008) Unsupervised natural experience rapidly alters invariant object representation in visual cortex. Science 321: 1502–1507. 18787171 10.1126/science.1160028 PMC3307055

[40] Li N, DiCarlo JJ (2010) Unsupervised Natural Visual Experience Rapidly Reshapes Size-Invariant Object Representation in Inferior Temporal Cortex. Neuron 67: 1062–1075. 20869601 10.1016/j.neuron.2010.08.029 PMC2946943

[41] Chen X, Han F, Poo MM, Dan Y (2007) Excitatory and suppressive receptive field subunits in awake monkey primary visual cortex (V1). Proceedings of the National Academy of Sciences of the United States of America 104: 19120–19125. 18006658 10.1073/pnas.0706938104 PMC2141918

[42] Rust NC, Schwartz O, Movshon JA, Simoncelli EP (2005) Spatiotemporal elements of macaque v1 receptive fields. Neuron 46: 945–56. 15953422 10.1016/j.neuron.2005.05.021

[43] Luenberger DG (1969) Optimization by vector space methods. New York, NY, USA: John Wiley & Sons, Inc., 326 pp.

[44] Löwdin PO (1950) On the Non-Orthogonality Problem Connected with the Use of Atomic Wave Functions in the Theory of Molecules and Crystals. The Journal of Chemical Physics 18: 365–375.

[45] Fan K, Hoffman AJ (1955) Some Metric Inequalities in the Space of Matrices. Proceedings of the American Mathematical Society 6: 111–116.

