# Supplementary material for: Slowness and Sparseness Have Diverging Effects on Complex Cell Learning
Source: PLoS Comput Biol. 2014 Mar 6;10(3):e1003468. doi: 10.1371/journal.pcbi.1003468 (PMC3945087; doi:10.1371/journal.pcbi.1003468)
Supplement: Figure S1 — Model complex cells derived with SFA fail to reproduce the small numbers of significant eigenvalues found empirically with STC analysis. We computed SFA filters on the quadratic feature space of the 100 lowest Fourier components of image patches sampled from the van Hateren image database [34]. As temporal transformation we applied a 2D translation with shift amplitudes drawn from a 2D uniform continuous distribution on to the data. We then applied the same analysis to the SFA filters as used in [42], [41]. We applied a sequence of 50000 Gaussian white noise pattern to the SFA filter. The filter responses were centered at their respective median and split in two firing rate sets, the excitatory from all positive responses (i.e. larger than median) and the inhibitory from the absolute value of all negative responses (i.e. smaller than median). The firing rates were then used to generate Poisson spike counts. Given spike counts and stimuli, we computed the spike triggered covariance (STC) for 100 different noise stimulus sets per SFA filter. The spectrum of eigenvalues (eigenspectrum) of the STC matrix of one cell recorded from V1 in an awake monkey [41] is shown in (A), the eigenspectrum of the STC of one SFA filter is shown in (B). To determine which eigenvectors are significant, we computed the STC with shuffled spike counts as control. The dashed lines correspond to mean 4.4 SD, which corresponds to a confidence interval of for Gaussian distributed eigenvalues. One clear difference is the number of significant eigenvectors. While for the V1 cell, only a few eigenvectors are significant, for the SFA model almost all eigenvectors are significant. The histogram of the number of significant excitatory and inhibitory eigenvectors is shown in (C) for the physiological data and in (D) for the SFA model. While the V1 cells have only few significant eigenvectors for all 130 recorded cells, the 980000 cells of the SFA model have on average 80 significant excitatory and inhi [file pcbi.1003468.s001.pdf]

Supplementary Material: Slowness and sparseness have diverging effects on complex cell learning

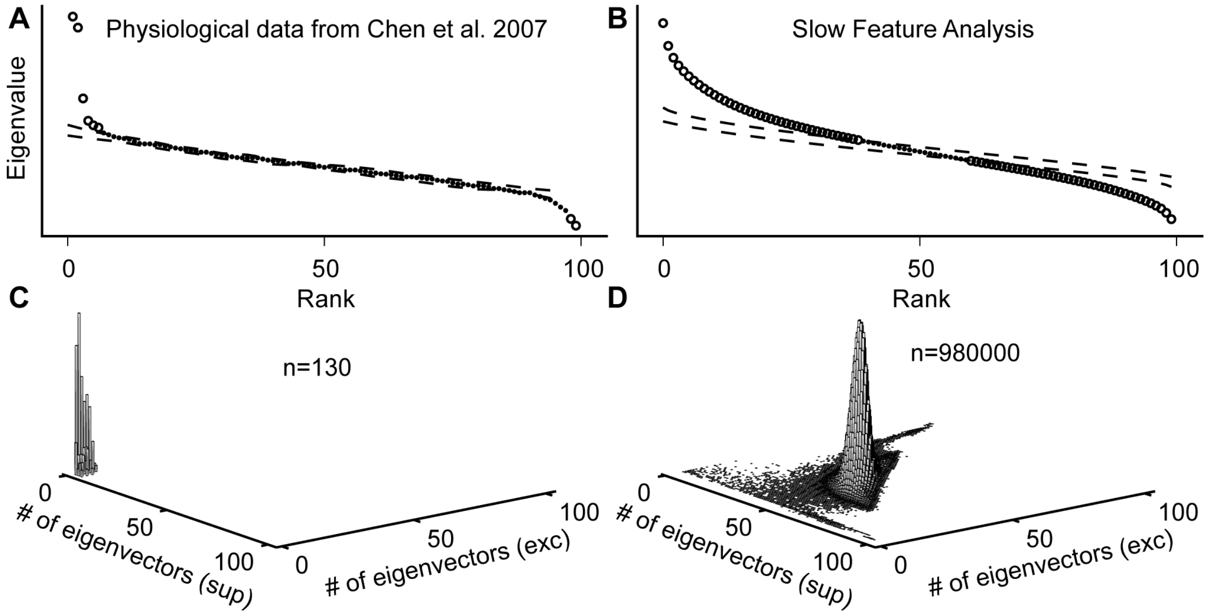

Figure S1. Model complex cells derived with SFA fail to reproduce the small numbers of significant eigenvalues found empirically with STC analysis. We computed SFA filters on the quadratic feature space of the 100 lowest Fourier components of 11×11 image patches sampled from the van Hateren image database [34]. As temporal transformation we applied a 2D translation with shift amplitudes drawn from a 2D uniform continuous distribution on $[-2:2]$ to the data. We then applied the same analysis to the SFA filters as used in [42,41]. We applied a sequence of 50000 Gaussian white noise pattern to the SFA filter. The filter responses were centered at their respective median and split in two firing rate sets, the excitatory from all positive responses (i.e. larger than median) and the inhibitory from the absolute value of all negative responses (i.e. smaller than median). The firing rates were then used to generate Poisson spike counts. Given spike counts and stimuli, we computed the spike triggered covariance (STC) for 100 different noise stimulus sets per SFA filter. The spectrum of eigenvalues (eigenspectrum) of the STC matrix of one cell recorded from V1 in an awake monkey [41] is shown in (A), the eigenspectrum of the STC of one SFA filter is shown in (B). To determine which eigenvectors are significant, we computed the STC with shuffled spike counts as control. The dashed lines correspond to mean $\pm$ 4.4 SD, which corresponds to a confidence interval of $P < 10^{-4}$ for Gaussian distributed eigenvalues. One clear difference is the number of significant eigenvectors. While for the V1 cell, only a few eigenvectors are significant, for the SFA model almost all eigenvectors are significant. The histogram of the number of significant excitatory and inhibitory eigenvectors is shown in (C) for the physiological data and in (D) for the SFA model. While the V1 cells have only few significant eigenvectors for all 130 recorded cells, the 980000 cells of the SFA model have on average 80 significant excitatory and inhibitory eigenvectors out of the 100 dimensions. The histogram bins with 0 entries were not plotted for clarity of the figure.
